# Hemi-precession maneuver to treat horizontal canal benign paroxysmal positional vertigo when the affected side is unilateral, bilateral or unknown

**DOI:** 10.3389/fneur.2025.1747706

**Published:** 2026-01-07

**Authors:** Marcello Cherchi

**Affiliations:** Department of Neurology, The University of Chicago, Chicago, IL, United States

**Keywords:** anatomy, benign paroxysmal positional vertigo, labyrinth, simulation, treatment, vestibular system

## Abstract

Benign paroxysmal positional vertigo (BPPV) is the most common cause of dizziness over the lifespan. The second most commonly affected semicircular canal in BPPV is the horizontal canal, which poses a diagnostic problem and two therapeutic problems. The diagnostic problem is the difficulty of determining the affected side(s) with confidence. This diagnostic uncertainly leads to the first therapeutic problem, which is that one may not know which side to treat. The second therapeutic problem is that because of the approximately co-planar configuration of the horizontal canals, most maneuvers that move otoliths on one side toward the utricle (which is desirable) will also move otoliths on the other side toward the ampulla (which is undesirable) — thus treatment of a given side will “undo” whatever might have been accomplished on the opposite side. Rather than working on the diagnostic dilemma, in this paper we focus on the therapeutic problems by devising a maneuver that leverages the vertical asymmetry of the horizontal canals in order to treat both sides simultaneously.

## Introduction

Benign paroxysmal positional vertigo (BPPV) is the most common cause of dizziness over the lifespan (1). Of all cases of BPPV, the horizontal canal variant is the second most common (after posterior canal). The proportion of cases for which it accounts has been debated, with most investigators citing rates of 8–17% (2), though some studies suggest the rate may be as high as 47.8% of cases (3).

Lateralization of the affected canal in horizontal canal benign paroxysmal positional vertigo (BPPV) is challenging. Some investigators find current methods are not superior to chance (4).

A large case series of 2,569 patients identified bilateral BPPV in 252 (9.8%) of cases (5). There are very few reported cases of confidently diagnosed bilateral horizontal canal BPPV (6, 7), and this is extraordinarily difficult to identify since BPPV of either horizontal canal can produce the same pattern of nystagmus. Nevertheless, given the overall statistics of bilateral BPPV cited earlier, it is plausible that cases of bilateral horizontal canal BPPV occur, and they may account for a higher proportion of BPPV than the rarity of the case reports suggests.

## Background

In view of the difficulty of lateralizing the affected side in (probable) unilateral horizontal canal BPPV, and given the likelihood that bilateral cases also occur, it would be desirable to develop a maneuver that effectively treats both sides.

One proposed solution was the Kurtzer hybrid maneuver (8), which the authors state, “consists of Appiani/Casani/Gufoni maneuvers combined into one fluid treatment.” The Gufoni maneuver (9) treats both geotropic and apogeotropic horizontal canal BPPV; the Appiani maneuver treats apogeotropic horizontal canal BPPV (10); the authors of the paper on the Kurtzer hybrid maneuver do not cite a specific paper for the “Casani maneuver,” which is odd since the main paper by Casani and colleagues pertains to treatment of anterior canal BPPV (11). In any case, there has been no literature on the Kurtzer hybrid maneuver since its publication, and we have not found this maneuver to be successful in our own clinical practice, though perhaps our technique is at fault.

The chief difficulty with treating bilateral horizontal canal BPPV, or treating unilateral horizontal canal BPPV whose side is unknown, is that the horizontal canals are mirror images of each other through the mid-sagittal plane. The approximately co-planar orientation of the horizontal canals means that in those positions (supine, prone, side-lying) that orient the horizontal canal approximately vertically, any gravitational vector that drives otoliths toward the utricle on one side will generally drive the otoliths toward the ampulla on the other side; thus if treatment is limited to logroll-type maneuvers, then treatment of one side will generally “undo” whatever had been accomplished by treating the other side.

The rare exception to this generalization would be a situation in which some otoliths are in the posterior section of the horizontal canal in one side (which in isolation would provoke geotropic direction-changing positional nystagmus), and some otoliths are in the anterior section of the horizontal canal on the other side (which in isolation would provoke apogeotropic direction-changing positional nystagmus). In this circumstance, a side-lying maneuver would either drive otoliths on both sides toward the respective ampullae, or would drive otoliths on both sides toward the respective utricles, and in either situation, the nystagmus generated from one side would oppose that generated from the other side, and they would at least partially cancel each other.

## Materials and methods

In designing a maneuver for treating bilateral horizontal canal BPPV it is useful to keep in mind that, despite the mirror-image configuration of the co-planar horizontal canals through the mid-sagittal plane, the target destination for the otoliths — namely, the utricle — is angled nearly perpendicular to the plane of the horizontal canals. This vertical (rostro-caudal) asymmetry can be leveraged in devising a treatment maneuver.

## Results

We used the BPPV Viewer simulator developed by Teixido and colleagues (12, 13) which is publicly available (https://bppvviewer.com, accessed 12/5/25) to develop and test a maneuver to treat benign paroxysmal positional vertigo (BPPV) involving one or both horizontal semicircular canals. We refer to this as a “hemi-precession” maneuver because the series of positions (Steps 2–9) resembles half of the path of precession of a spinning top.

This maneuver assumes that the patient has been diagnosed with horizontal canal BPPV, and only horizontal canal BPPV. Most clinicians reach this conclusion after observing direction-changing positional nystagmus (either geotropic or apogeotropic) during the side-lying maneuvers, or (less commonly) during the Dix-Hallpike maneuvers.

In the legend for each Figure we include the BPPV Viewer position parameters so that readers can replicate this simulation.

### Step 1

**Figure 1:**
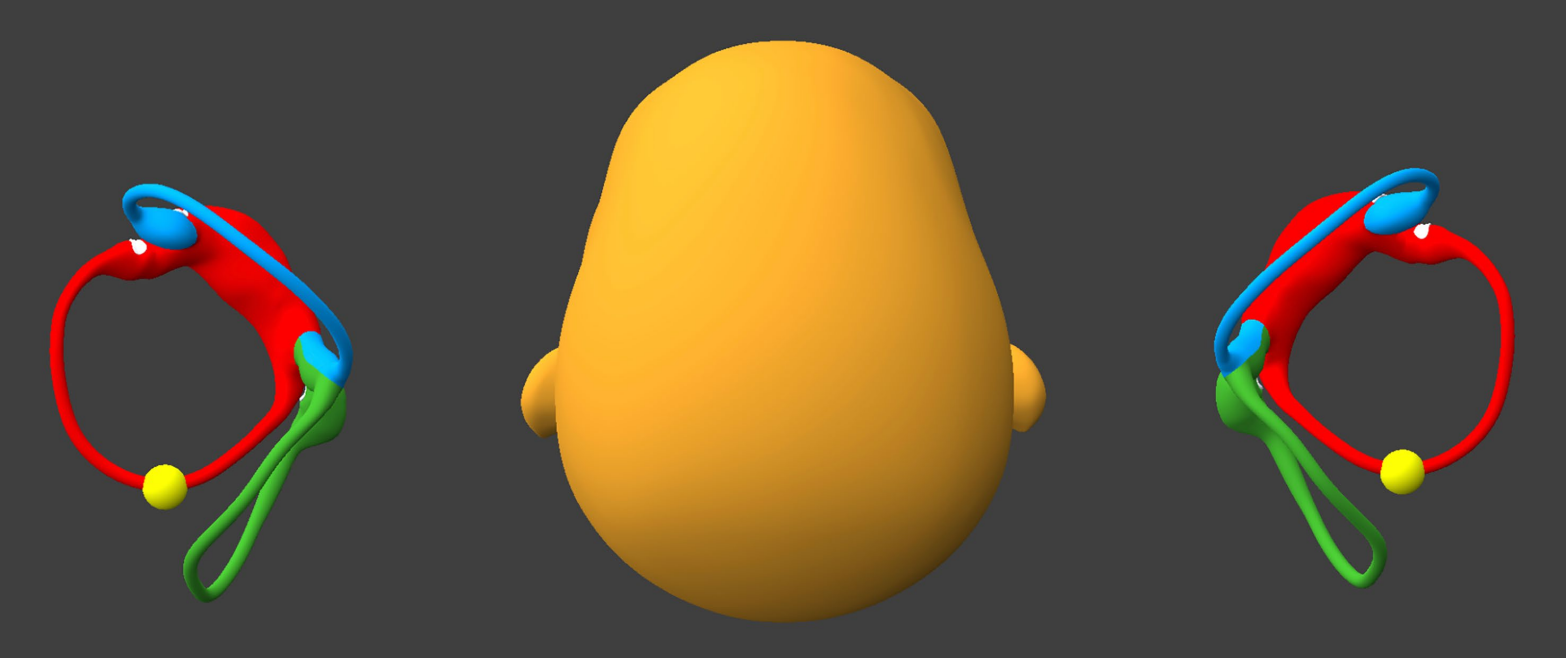
Alternate side-lying positions until the direction-changing positional nystagmus is consistently geotropic. BPPV Viewer parameters: X = 285°, Y = 180°, Z = 0°. See text for details.

Preparatory to this maneuver, it is important to alternate between the right side-lying position and the left side-lying position, pausing for 30–60 s in each position, until the resulting direction-changing positional nystagmus is consistently geotropic; the goal of this is to convert any possible apogeotropic direction-changing positional nystagmus (which reflects that otoliths are in the anterior segment of the horizontal canal) into geotropic direction-changing positional nystagmus (which reflects that otoliths are in the posterior segment of the horizontal canal). When the direction-changing positional nystagmus is consistently geotropic, then this suggests that the otoliths have coalesced in the posterior-most segment of the horizontal canal(s), as shown in Figure 1.

### Step 2

**Figure 2:**
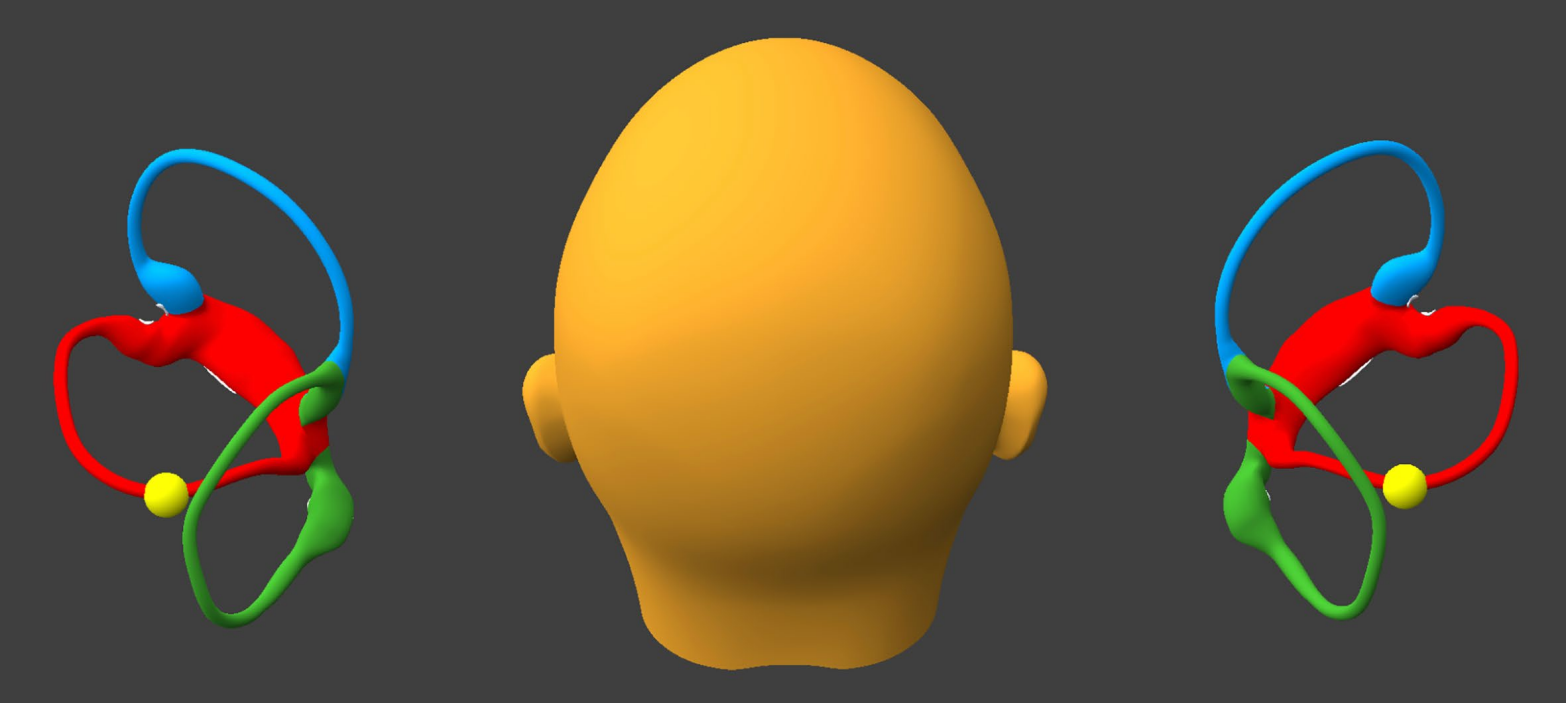
Incline the head backward about 30°. BPPV Viewer parameters: X = 330°, Y = 180°, Z = 0°. See text for details.

The patient sits up, but inclines the head backward about 30° (like the “lean” position of the “bow and lean” test), as shown in Figure 2. This will keep the otoliths (whether on one, or the other, or both sides) in the posterior-most segment of the horizontal canal(s).

### Step 3

**Figure 3:**
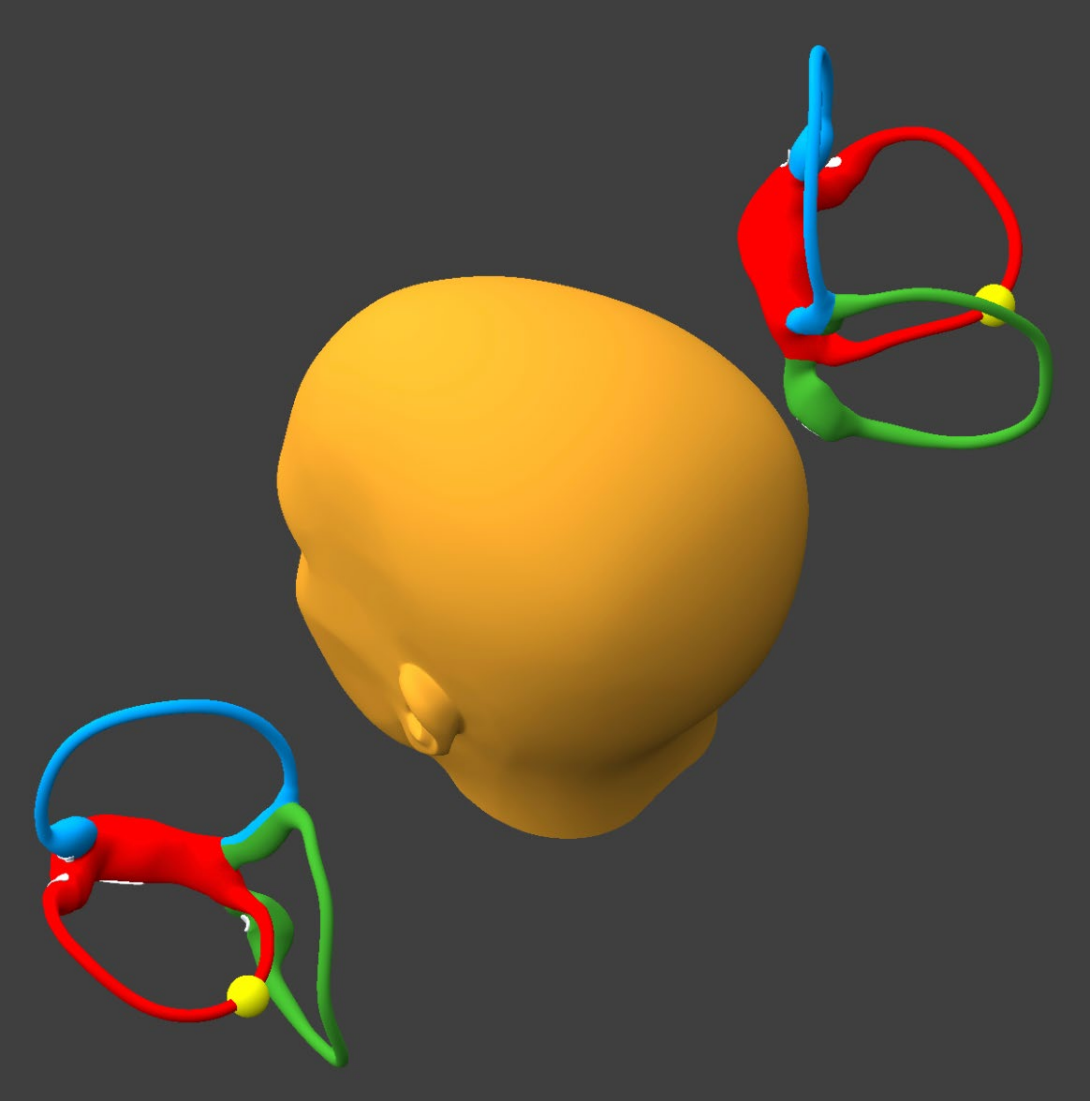
Tilt the head backward and leftward about 30°. BPPV Viewer parameters: X = 330°, Y = 225°, Z = 0°. See text for details.

While remaining seated upright, the patient tilts the head backward about 30° and leftward about 30°, as shown in Figure 3.

- If at the beginning of this step otoliths are present in the posterior-most segment of the right sided horizontal canal, then they will proceed toward the singular crus.
- If at the beginning of this step otoliths are present in the posterior-most segment of the left sided horizontal canal, then they will progress part-way toward the lateral-most segment of the left sided horizontal canal.

### Step 4

**Figure 4:**
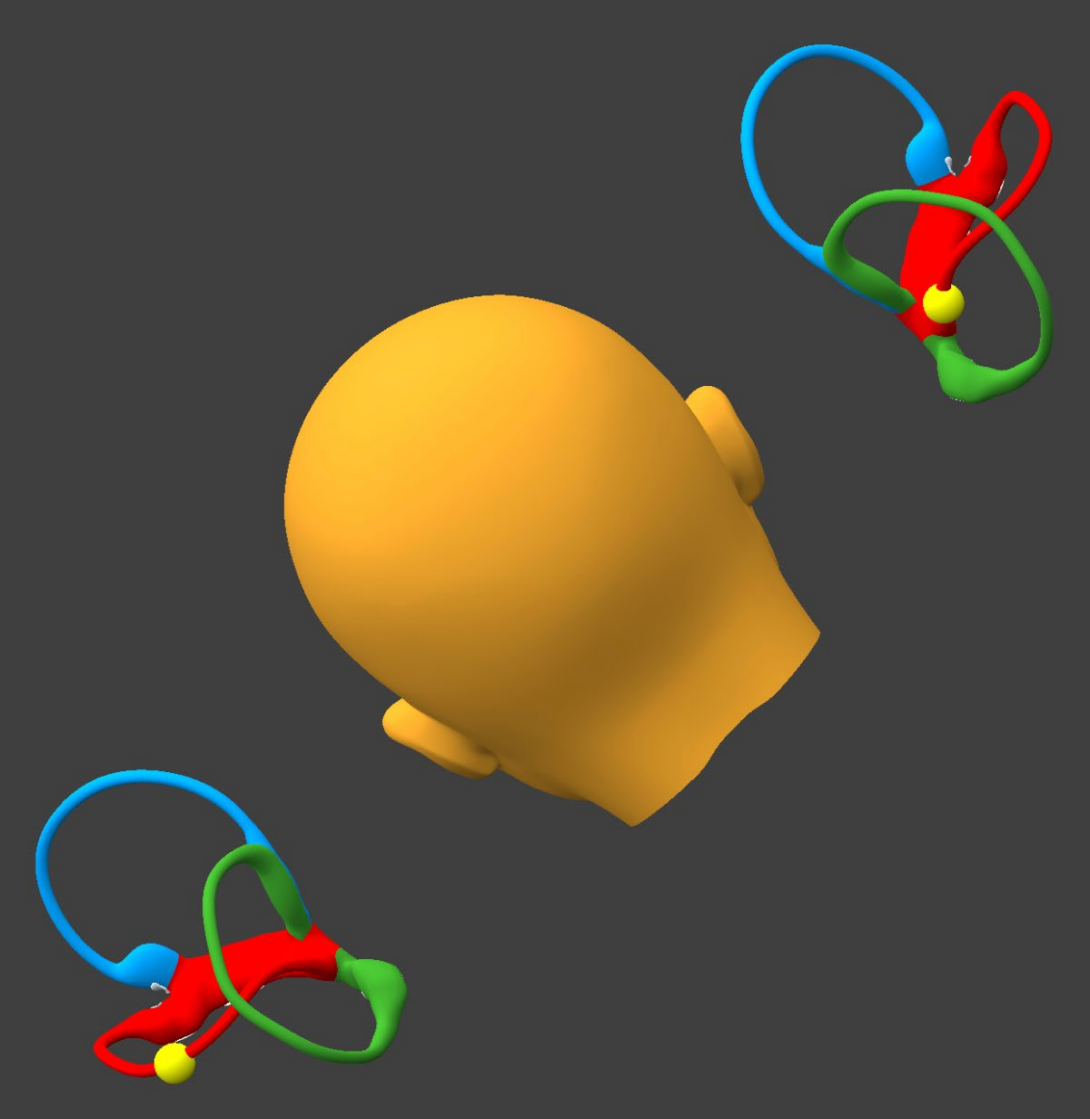
Tilt the head laterally to the left about 30°. BPPV Viewer parameters: X = 0°, Y = 180°, Z = 30°. See text for details.

While remaining seated upright, the patient tilts the head laterally toward the left (flexing the neck directly laterally), as shown in Figure 4.

- If at the beginning of this step otoliths are present in the right horizontal canal near the singular crus, then they will enter the singular crus, pass through it, and migrate inferiorly into the right utricle.
- If at the beginning of this step otoliths are present in the left horizontal canal, then they will progress to, and reach, the lateral-most segment of the left sided horizontal canal.

### Step 5

**Figure 5:**
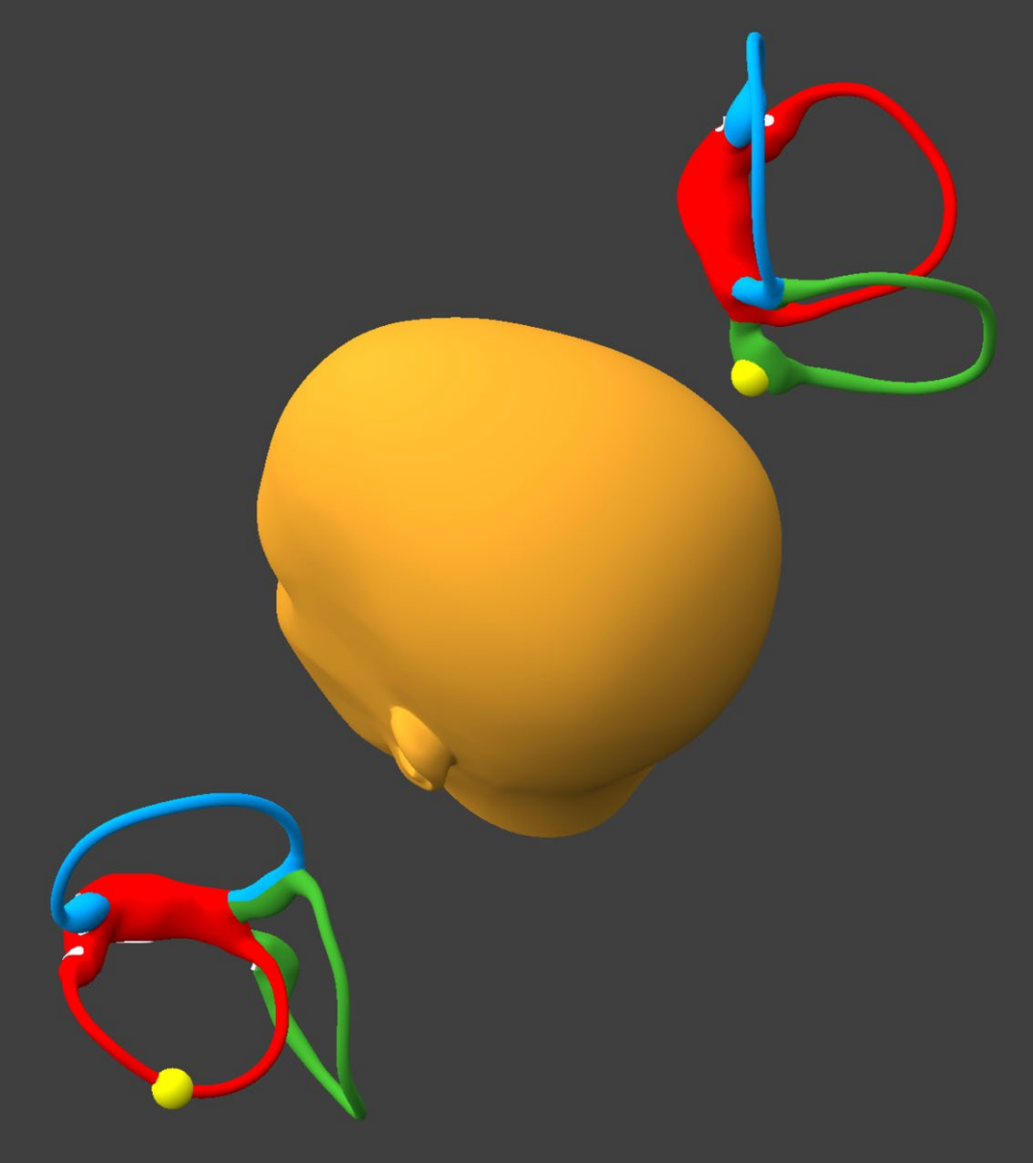
Tilt the head backward and leftward, as in Step 3. BPPV Viewer parameters: X = 330°, Y = 225°, Z = 0°. See text for details.

The position shown in Figure 5 is the same as in Step 3. However:

- If at the beginning of this step otoliths are present in the right utricle, then they will remain sequestered there.
- If at the beginning of this step otoliths are present in the left horizontal canal, then during this step they will move toward the utricle and by the end of this step they will come to rest in between the lateral-most and posterior-most segments of that canal.

### Step 6

**Figure 6:**
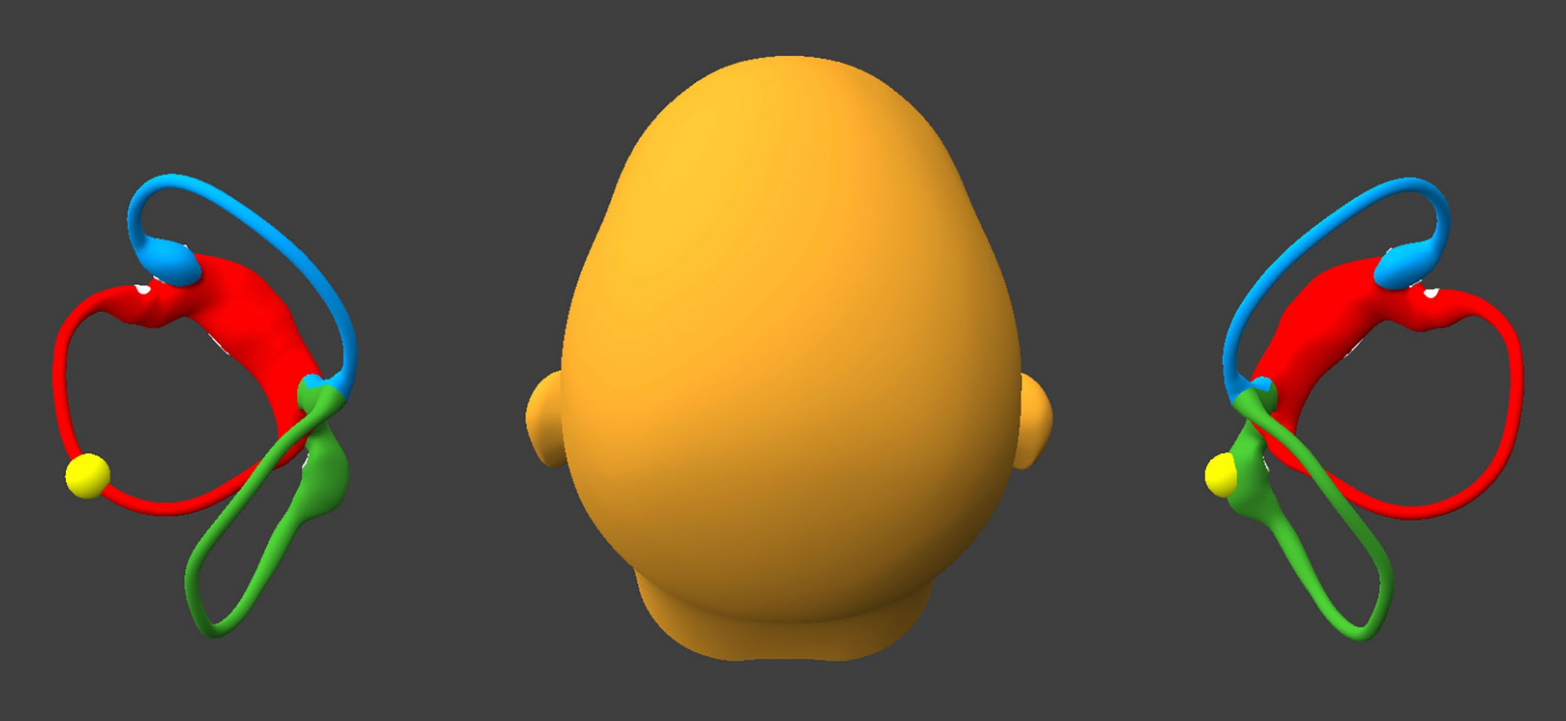
Tilt the head backward about 30°, as in Step 2. BPPV Viewer parameters: X = 330°, Y = 180°, Z = 0°. See text for details.

The position shown in Figure 6 is the same as in Step 2. However:

- If at the beginning of this step otoliths are present in the right utricle, then they will remain sequestered there.
- If at the beginning of this step otoliths are present in the left horizontal canal, then they will move toward the utricle, and by the end of this step they will come to rest in the posterior-most segment of that canal.

### Step 7

**Figure 7:**
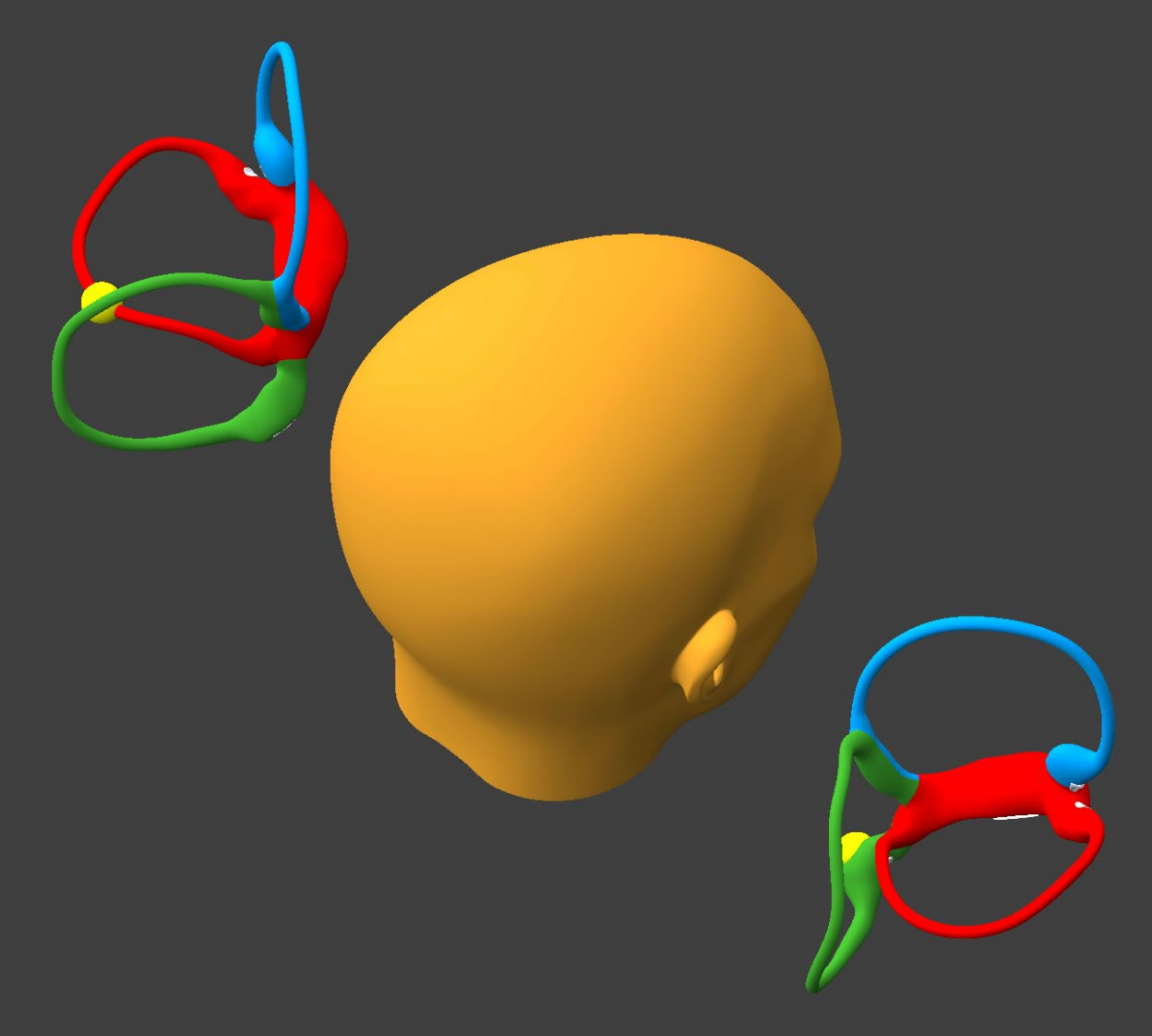
Tilt the head backward and rightward about 30°. BPPV Viewer parameters: X = 330°, Y = 150°, Z = 0°. See text for details.

While remaining seated upright, the patient tilts the head backward about 30° and rightward about 30°, as shown in Figure 7.

- If at the beginning of this step otoliths are present in the right utricle, then they will remain sequestered there.
- If at the beginning of this step otoliths are present in the left horizontal canal, then they will progress toward the singular crus.

### Step 8

**Figure 8:**
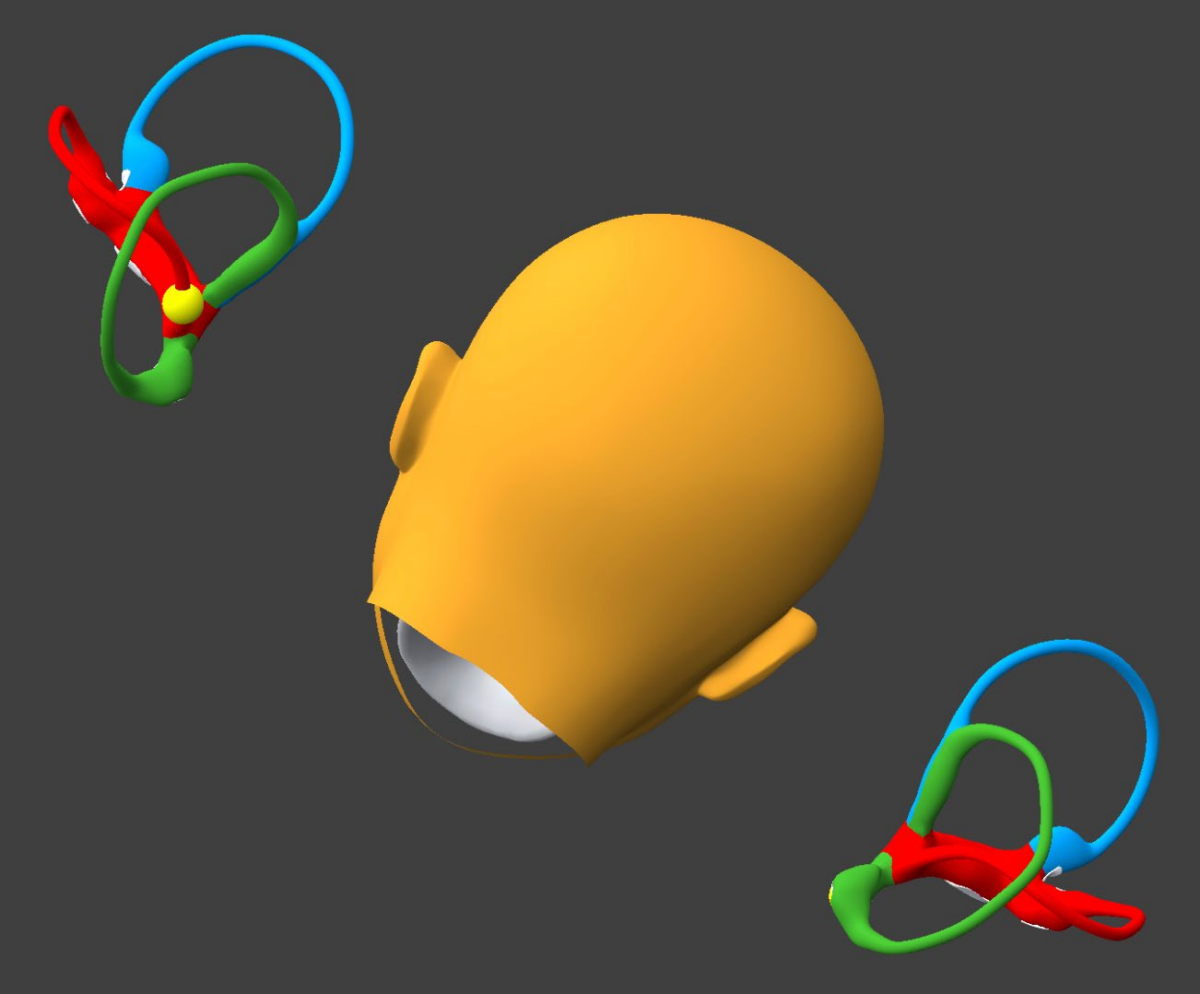
Tilt the head laterally to the right about 30°. BPPV Viewer parameters: X = 0°, Y = 180°, Z = 330°. See text for details.

While remaining seated upright, the patient tilts the head laterally toward the right (flexing the neck directly laterally), as shown in Figure 8.

- If at the beginning of this step otoliths are present in the right utricle, then they will remain sequestered there.
- If at the beginning of this step otoliths are present in the left horizontal canal near the singular crus, then they will enter the singular crus, pass through it, and migrate inferiorly into the left utricle.

### Step 9

**Figure 9:**
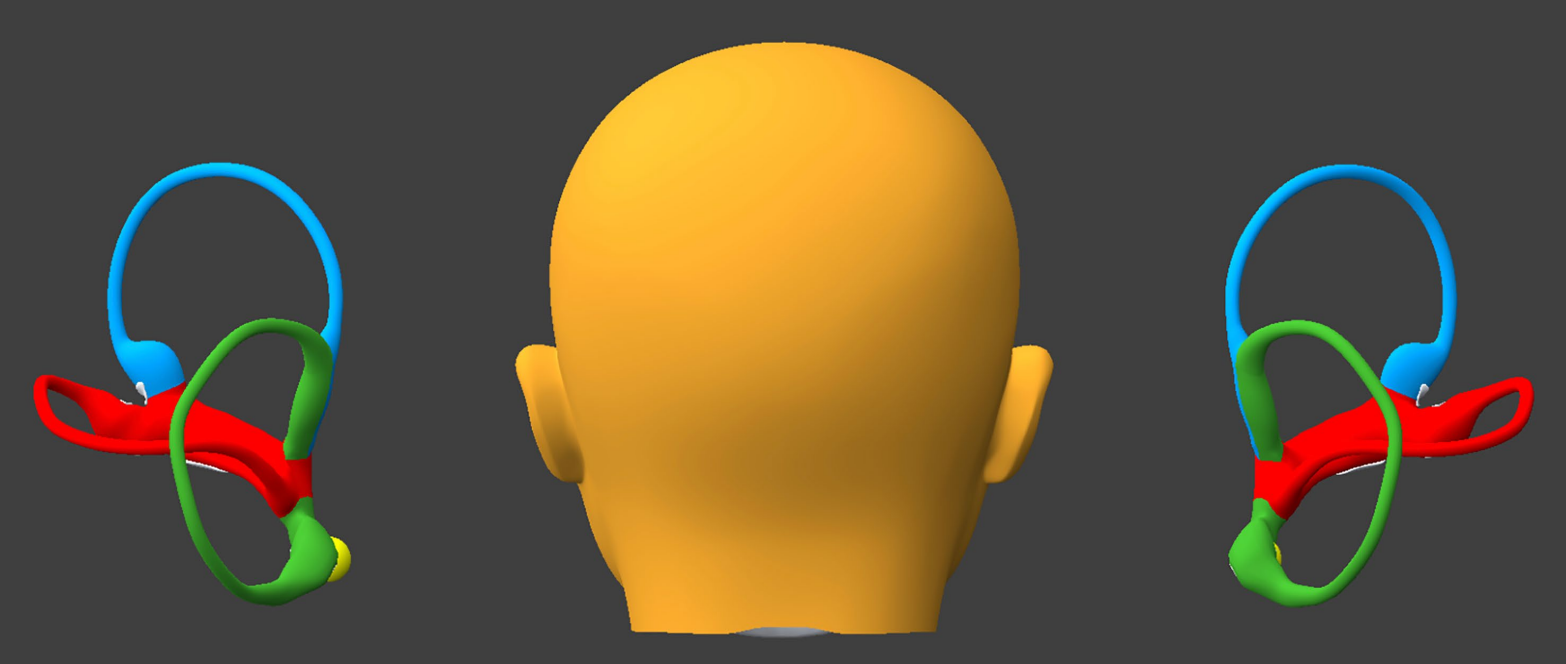
Resume an upright, neutral posture. BPPV Viewer parameters: X = 0°, Y = 180°, Z = 0°. See text for details.

The patient resumes an upright seated posture with the head in a neutral position, as shown in Figure 9. At the point any otoliths that had been in either horizontal canal will be in the respective utricle.

An animated video illustrating this maneuver is provided in the Supplementary materials.

## Discussion

Horizontal canal benign paroxysmal positional vertigo poses a diagnostic dilemma (correct lateralization) and therapeutic challenges. In this paper we have bypassed the diagnostic dilemma by developing a maneuver intended to treat both horizontal canals simultaneously by leveraging their vertical asymmetry.

There are several advantages to this maneuver.

First, it should treat BPPV of both horizontal canals.

Second, since it does not involve head positions in which the head is angled downward (such as the Dix-Hallpike, or other positions in which the crown is lower than the foramen magnum), there is no opportunity for otoliths to reflux from the utricle into the common crus — in other words, the risk of canal conversion (passage of otoliths into the anterior or posterior canals) and reflux (of otoliths back into the horizontal canal) should be extremely low.

There are also disadvantages to this maneuver.

First, since the plane of the horizontal canal is never perpendicular to earth-vertical, the force vector along the lumen of the canal is always less than maximal; consequently, it will take longer for otoliths to reach the most dependent section of the canal in any given position. Practically, this means that any given position of the maneuver will need to be held for longer, though how much longer remains to be established.

Second, some of the head-on-neck positions may be difficult for patients with limited cervical range of motion. However, this disadvantage is not unique to this maneuver.

Third, the maneuver is complex in the sense that it involves 9 steps. This may limit the degree to which it would be adopted in practice, and could also lower the likelihood of its use as a home treatment maneuver.

Fourth, this maneuver would require otoliths to be mobile (canalolithiasis), and thus will not work for cupulolithiasis.

Finally, there is a disadvantage to this study, in that is uses a simulator. Future study should involve human patients, and should be tested against maneuvers with comparable goals, such as the Kurtzer hybrid maneuver (8).

## Data Availability

The original contributions presented in the study are included in the article/Supplementary material, further inquiries can be directed to the corresponding author.
